# An Improved UNet++ Model for Congestive Heart Failure Diagnosis Using Short-Term RR Intervals

**DOI:** 10.3390/diagnostics11030534

**Published:** 2021-03-16

**Authors:** Meng Lei, Jia Li, Ming Li, Liang Zou, Han Yu

**Affiliations:** 1 School of Information and Electrical Control Engineering, China University of Mining and Technology, Xuzhou 221116, China; lmsiee@cumt.edu.cn (M.L.); TS18060175P31@cumt.edu.cn (J.L.); liming@cumt.edu.cn (M.L.); 2 School of Computer Science and Engineering (SCSE), Nanyang Technological University (NTU), Singapore 639798, Singapore; han.yu@ntu.edu.sg

**Keywords:** congestive heart failure, short-term RR intervals, UNet++

## Abstract

Congestive heart failure (CHF), a progressive and complex syndrome caused by ventricular dysfunction, is difficult to detect at an early stage. Heart rate variability (HRV) was proposed as a prognostic indicator for CHF. Inspired by the success of 2-D UNet++ in medical image segmentation, in this paper, we introduce an end-to-end encoder-decoder model to detect CHF using HRV signals. The developed model enhances the UNet++ model with Squeeze-and-Excitation (SE) residual blocks to extract deep features hierarchically and distinguish CHF patients from normal subjects. Two open-source databases are utilized for evaluating the proposed method, and three segment lengths of intervals between successive R-peaks are employed in comparison with state-of-the-art methods. The proposed method achieves an accuracy of 85.64%, 86.65% and 88.79% when 500, 1000 and 2000 RR intervals are utilized, respectively. It demonstrates that HRV evaluation based on deep learning can be an important tool for early detection of CHF, and may assist clinicians in achieving timely and accurate diagnoses.

## 1. Introduction

Congestive heart failure (CHF) is the terminal stage of a variety of cardiovascular diseases, such as hypertension, coronary heart disease, and valvular heart disease [1]. Frequently-reported symptoms include left ventricular hypertrophy and left ventricular dilation, which may lead to neuroendocrine disorders and circulatory dysfunction [2]. If left untreated or not treated properly, heart failure will gradually worsen over time. According to epidemiological surveys around the world, for every 100 people, 3–5 people have different levels of heart failure. However, diagnosis is often difficult, especially for early diagnosis. Early diagnosis of CHF is able to slow down the progression prior to adverse events and improve the patients’ chance of survival [3]. Moreover, the mortality rate in CHF patients within 5 years is as high as 50%, which makes it a major public health challenge [4]. Therefore, CHF diagnosis with high objectivity and reliability is highly desired.

Electrocardiogram (ECG), which contains abundant information of cardiac activities, is commonly utilized for heart rhythm analysis in hospitals [5]. However, manual ECG signal analysis is time-consuming, and the quality of diagnostic results depends on the clinician’s expertise [6]. To address the limitations of manual ECG analysis, various automated CHF detection algorithms have been proposed. For instance, Gotsman et al. [7] found that QRS-T angle is relatively stable in patients with heart failure, and widening of the QRS-T angle has predictive value. The equal frequency in amplitude and equal width in time (EFiA-EWiT) discretization method was proposed to extract features from ECG signals, and a linear regression model was used to differentiate CHF from normal sinus rhythm (NSR) patterns [6]. Acharya et al. developed a fully automatic CHF diagnosis model based on an 11-layer convolutional neural network which achieved an accuracy of 98.97% on a data set from PhysioBank [8].

RR interval, the time elapsed between successive R-peaks extracted from ECG signals, may contain useful information about heart diseases [9]. Previous studies show that heart rate variability (HRV), a phenomenon of the variation of heart rate in the time intervals, can serve as a biomarker for disease severity in patients with CHF [10]. Long-term HRV signals have been employed for CHF detection. Using Poincare plot, Tushar et al. [11] studied the differences of lag-response in CHF patients and normal subjects, and found that curvilinearity was lost in patients with CHF after exploring sequences up to 50,000 beats. A multistage classification method using non-equilibrium decision-tree-based support vector machine (DT-SVM) was proposed for risk assessment based on HRV [12]. Fifty four classical measures and 126 dynamic indices were extracted to detect and quantify CHF patients. Isler et al. [13] applied genetic algorithm (GA) to select the best features from the combination of standard HRV measures and wavelet entropy measures. They further employed the k-nearest neighbor classifier to distinguish 29 CHF patients from 54 healthy subjects.

Despite providing promising performance, existing methods always require long-term data which are difficult to acquire. For example, Yu et al. [14] proposed bispectral analysis and genetic algorithm for CHF detection based on long-term (24-h) HRV. Although their proposed method achieved a high accuracy of 96.38%, acquiring 24-h HRV data is not easy outside of the hospital environment, especially with mobile devices. Recently, various machine learning-based CHF detection methods have been developed with different lengths of short-term RR intervals and achieved inspiring performance. Liu et al. [15] proposed a multiscale entropy method to classify normal subjects from CHF patients and achieved accuracy of 85.5% and 85.6% when 1000 and 2000 RR intervals are used, respectively. Wang et al. [16] combined handcrafted features and deep-learning features, and utilized an ensemble classifier for CHF detection. Three types of RR segment length (*N* = 500, 1000 and 2000) were used to evaluate their proposed method with accuracy of 83.84%, 87.54%, and 85.71%, respectively.

Recently, deep learning algorithms are widely used in medical signal analysis and have achieved remarkable results [17,18]. Deep neural networks are able to automatically learn discriminative features from raw data. They have shown enormous potential in analyzing heterogeneous data and great generalization ability. For example, Chen proposed a sparse auto-encoder-based deep learning model in CHF detection using RR intervals [19]. Li et al. [20] combined deep neural network and distance distribution matrix to identify CHF and achieved an accuracy of 81.85%. Wang et al. [21] used long short-term memory (LSTM) deep network to detect CHF based on RR intervals and achieved 82.51%, 86.68% and 87.55% accuracy on *N* = 500, 1000 and 2000 RR intervals, respectively.

Although the above-mentioned studies have made significant progress, there are still two major limitations. Firstly, mainstream CHF detection algorithms are mostly based on handcrafted features. However, such approaches are not robust and rely too much on expert experience. To extract effective temporal features from HRV data, some researchers integrated time-domain, frequency-domain and nonlinear features together to form feature vectors [16], which is time-consuming and error-prone. In addition, such features generally are task-specific and lack generality. Meanwhile, errors in feature extraction process may be propagated to later stages, which negatively impact the detection performance. Although a few deep learning-based strategies were proposed for exploring the information of RR intervals, the performance is not satisfactory in comparison with approaches based on time-series ECG signals. Secondly, patients with mild CHF might be misclassified with healthy controls because the classification boundaries between them are not clear. Existing studies show that there is still great room for improvement, especially for mild CHF patients detection.

To address these problems, this paper presents an encoder-decoder model for CHF detection based on short-term RR intervals. The main contributions are summarized as follows:

1. This paper proposes a novel strategy to extract the features from short-term RR intervals, at most with the length of 2000. The end-to-end decision support system extracts deep features automatically from raw data, which not only improves the generalization but also avoids error propagation and information reduction.
2. Inspired by the success of UNet++ in computer vision tasks, this paper presents a novel strategy to classify patients with CHF and normal subjects via improved 1-D UNet++. To the best of our knowledge, no prior work on ECG analysis has employed the 1-D UNet++ network. In addition, to increase the sensitivity to informative features, the squeeze and excitation block are integrated [22] and combined with residual networks [23]. The improved 1-D UNet++ has achieved state-of-the-art accuracy of 88.79% on two publicly available data sets with mild CHF patients.

## 2. Materials and Methods

**Figure 1:**
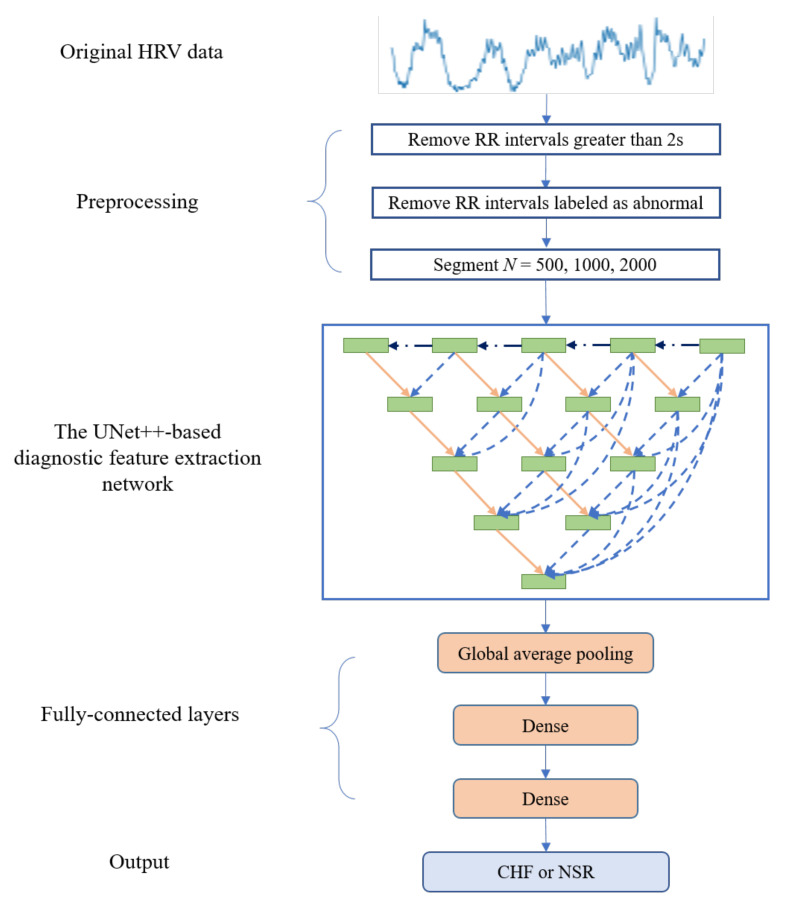
An overview of the method used in this work.

The proposed framework, consisting of data preprocessing, feature-extraction based on the improved UNet++ and classification based on fully connected layers, is illustrated in Figure 1. The details of each parts are discussed in the following sub-sections.

### 2.1. Dataset and Preprocessing

**Figure 2:**
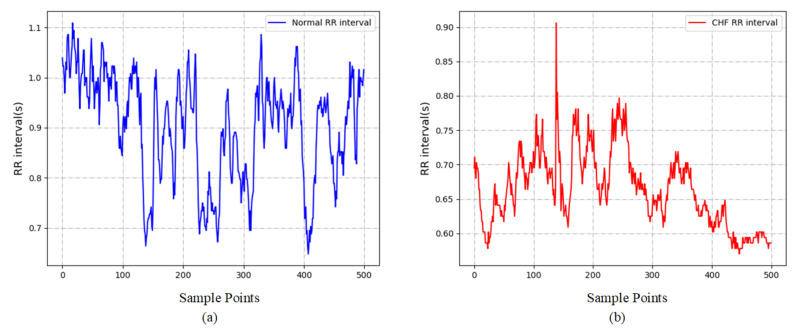
Two demonstrative examples for the RR intervals corresponding to (**a**) normal subjects and (**b**) CHF patients.

All data used in this study are obtained from PhysioBank [24], an open-access archive of physiological signals. For normal subjects, the normal sinus rhythm RR interval database (NSR-RR) is used. This database includes 54 long-term RR interval recordings from normal subjects aged from 29 to 76. The congestive heart failure RR interval database (CHF-RR) comprises 29 subjects with CHF (NYHA classes I, II and III) aged from 34 to 79. The raw ECG signals of both NSR-RR and CHF-RR databases were digitized at 128 samples per second, and the beat information was annotated through automated analysis with manual correction provided by PhysioNet [24]. RR interval is the time interval between successive cardiac cycle and has attracted wide attention for its potential to diagnose CHF. The pre-processing procedure for the RR intervals in this paper includes two steps:

1. Each beat in original ECG signals was annotated as normal (labeled as ‘N’) or abnormal (usually caused by the ectopic beats). The RR intervals marked as abnormal are removed to avoid the negative effects on analysis of HRV. Meanwhile, the RR intervals longer than 2 s are also removed to avoid the error accumulated in the precedent peak detection.
2. We split the ECG signal of each subject into multiple RR segments. This approach not only augments the data set, but also avoids the problem of time-consuming process in long-term HRV signal analysis [25]. To compare the results with other studies, the signals are divided into 500, 1000 and 2000 RR intervals, as in [15,16]. Table 1 shows the details for these two datasets used in this study. Two demonstrative examples of the signals corresponding to NSR and CHF with 500 RR intervals are shown in Figure 2.

### 2.2. Proposed Network Architecture

The encoder-decoder architecture has become increasingly popular in feature extraction due to its high flexibility and superiority. UNet++ is a useful new variant of UNet, proposed by Zhou et al. [26,27]. A series of nested dense convolutional blocks connect the encoder and decoder in UNet++, which can narrow and fill the information gap between the feature maps of the encoder and decoder prior to fusion. In this study, given that the CHF data is one-dimension, the 1-D variant of classical UNet++ model is developed to explore the pathological variations of CHF based on RR interval recordings. The 1-D UNet++ is able to capture valuable details of the HRV signals effectively since high dimension feature maps from the encoder part are gradually enriched prior to fusion with the corresponding pathologically rich feature maps from the decoder part.

**Figure 3:**
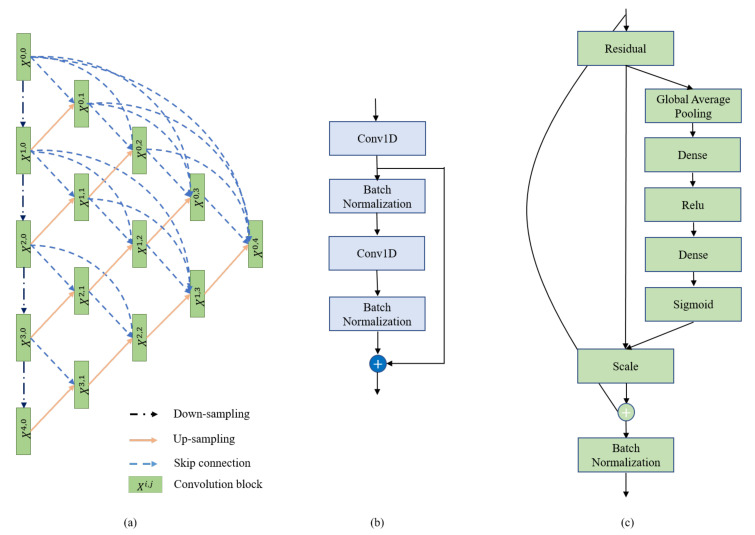
Structure of (**a**) the overall UNet++ network structure, (**b**) residual module and (**c**) convolution block.

RR intervals of different lengths, after being processed, are utilized as the input of the network. As shown in Figure 3a, the 1-D UNet++ structure consists of convolution blocks, down-sampling and up-sampling modules. Each black arrow denotes a down-sampling step which is implemented using a maximum pooling operation with kernel size of 2 and stride of 1. This window and stride configuration halves the size of the feature map. Such down-sampling can effectively extract features of the input and enhance robustness to noise by condensing features. The orange arrows represent the opposite operation, namely up-sampling, doubling the size of the feature maps. Up-sampling is the part that restore characteristics which is the highly effective expression form of the input data.

As shown in Figure 3a, we assume $x^{i,j}$ denotes the output of node $X^{i,j}$, where *i* represents *i*th down-sampling layer along the encoder way and *j* represents the *j*th convolution layer along the skip pathway. The accumulation of feature maps by $x^{i,j}$ can be defined as:

(1) $$x^{i,j}=\left\{\begin{array}{cc} H(x^{i-1,j}), & j=0\\ H(\left[{\left[x^{\left(i,k\right)}\right]}_{k=0}^{j-1},u\left(x^{i+1,j-1}\right)\right]), & j>0 \end{array}\right.$$

where $H\left(\cdot \right)$ denotes a 1-D convolution operation combined with an activation function, $u\left(\cdot \right)$ presents an up-sampling layer, and $\left[\cdot \right]$ is the concatenation operation. Generally, nodes at level j=0 receive only one input from the previous layer of the encoder while nodes at level j>0 receive j+1 inputs from both the skip connections and the up-sampling layer.

Residual modules are incorporated in the convolution unit, which facilitates convergence of the proposed deep model [28]. As can be seen in Figure 3b, the original residual module contains 1-D convolution layers (Conv1D) [29] and Batch Normalization (BN) layers [30] which are implemented alternately. The output of the residual module is generated by adding the outputs of the first Conv1D layer and the second BN layer. Inspired by the effectiveness of squeeze-and-excitation features on image object classification, 1-D squeeze-and-excitation (SE) residual modules are employed as convolution units in UNet++, as shown in Figure 3c. The SE residual modules can adaptively recalibrate residual feature maps within each feature channel by explicitly modeling interdependency between channels. It can enhance the representational power of modules throughout the network [22].

### 2.3. Model Structure and Parameters

In this work, a deep learning model is built to perform CHF detection using RR interval recordings. As shown in Figure 3a, the dimension of output to encoding is 1/16 of the input size. In this work, normal and CHF recordings, each with 500, 1000 or 2000 samples, are changed to 512, 1008 and 2000 using zero padding, respectively and then are fed into the input layer of this model. A global average pooling [31] is used to summarize the information from all the feature maps. Finally, an automatic prediction is provided by learning of these feature maps in the dense layer.

The signals from 83 subjects are split into 10 parts and ensure that all the signals from each subject are in one part. 10-fold cross validation is employed to evaluate the robustness of the proposed model. For each iteration, 9 parts are used for training and the remaining one part is used for testing. The method is repeated 10 times by shifting the testing part. The test set consists of the RR intervals from the subjects who are not used in the training process, and therefore reduces the possibility of over-fitting. We empirically set the batch size as 16 and the number of training epochs as 70 (*N* = 500, 1000 and 2000 length RR intervals). During the training process, we first set the initial learning rate as $10^{-4}$, and update its value as 0.1 times of the original one when the validation loss stops improving within 5 epochs. Adam optimizer and mean squared error loss function are applied. The loss function is defined as:

(2) $$L\left(y,p\right)=\frac{1}{N}\sum_{i=1}^{N}{\left(y_{i}-p_{i}\right)}^{2}$$

where *N* is the number of samples, $y_{i}$ and $p_{i}$ is the true label and the prediction result of the *i*th sample.

### 2.4. Performance Measures

To evaluate the performance of the proposed method and make a fair comparison, we employ four widely used evaluation metrics, including accuracy, recall, precision and F1-score [32,33].These four indicators are widely considered to be the most informative for evaluating the performance of classifiers and convenient for calculation. All these evaluation metrics can be calculated by following formula:

(3) $$Accuracy=\frac{TP+TN}{TP+FP+TN+FN}$$

(4) $$Recall=\frac{TP}{TP+FN}$$

(5) $$Precision=\frac{TP}{TP+FP}$$

(6) $$F1-score=\frac{2}{\left[\frac{1}{Precision}+\frac{1}{Recall}\right]}$$

Here, true positives (TP) is the number of CHF segments correctly classified as CHF group; false positives (FP) is the number of NSR segments wrongly classified as CHF group. True negatives (TN) associates with the number of NSR segments correctly classified as NSR group; false negatives (FN) is the number of CHF segments wrongly classified as NSR group.

## 3. Results

### 3.1. 10-Fold Cross Validation Performance

In the traditional cross validation, the RR intervals from one subject may appear in both training and test set. However, the similarity between these signals will lead to information leakage and result in overoptimistic performance. Considering the practical application of classification system to diagnosis unknown subjects, the dataset is split into training and test set in terms of the subjects in this study. The RR intervals of one subject appear in either the training set or the test set in each iteration.

**Figure 4:**
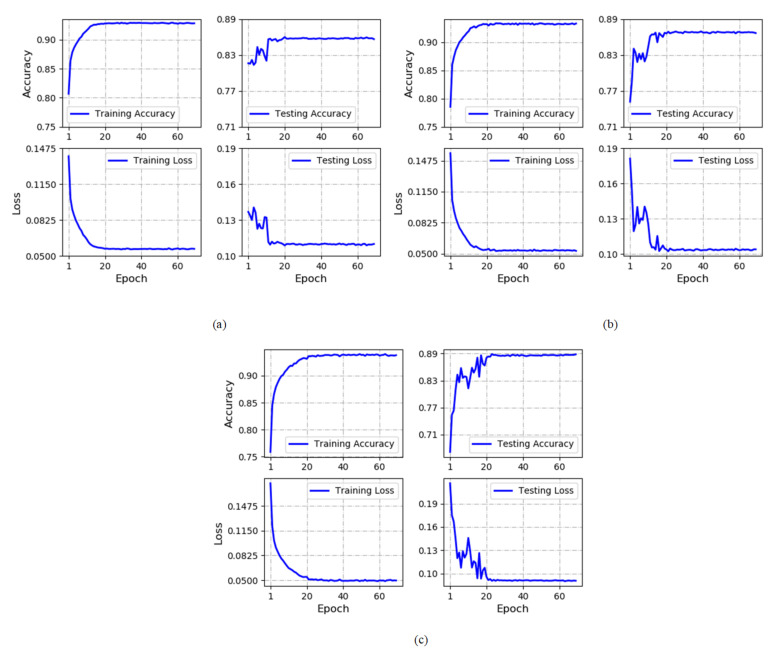
Performance graphs of the proposed model. (**a**) The RR segment length *N* = 500; (**b**) The RR segment length *N* = 1000; (**c**) The RR segment length *N* = 2000.

The training details in terms of the accuracy and the loss against each epoch are presented in Figure 4. The solid line is the average of the performance across 10-folds. In the training phase, the model reaches convergence within a short time (mostly 20 epochs). The fluctuations of the performance on the test set is relative small, which demonstrates that the developed model generalizes well on separate dataset. Specifically, the training accuracy converges at 92.82%, 93.36% and 93.79% when 500, 1000 and 2000 RRs are employed respectively. The corresponding test accuracy converges at 85.64%, 86.65% and 88.79%.

### 3.2. Comparison with Different Network Architectures

Inception was a deep learning network model, designed by Christian Szegedy and others [34]. It is not only able to efficiently reduce the number of parameters, but also capable of increasing the expression ability of the network by introducing more linear mappings. In this study, the CHF detection results of the improved UNet++ are compared with those of introducing the inception modules on account of the excellent performance of Inception. In addition, the performance of the UNet++ model with or without SE modules are evaluated for illustrating its effect.

The results and comparisons of three RR segment length types (*N* = 500, 1000 and 2000) for different methods are listed in Table 2. The testing accuracy of the proposed UNet++ model among all the RR intervals length is over 85%. The best classification performance is yielded when 2000 RR intervals from each subject are employed, reaching an accuracy of 88.79%. These outperforming results indicate that the proposed model is effective for CHF detection.

It is worth noting that the improved UNet++ with SE residual units consistently outperforms other network structures when three different lengths of RR intervals are employed, in terms of Recall, Precision, F1-score and Accuracy. The mean accuracy of the plain 1-D UNet++ across 10-fold across validation is 82.57%, 81.84% and 82.69% for *N* = 500, 1000 and 2000, respectively, whereas that of improved 1-D UNet++ is 85.64%, 86.65% and 88.79%, respectively. By introducing SE residual blocks to the original 1-D UNet++, the mean accuracy is increased by 3.07% 4.81% and 6.10% when *N* = 500, 1000 and 2000, respectively.

**Figure 5:**
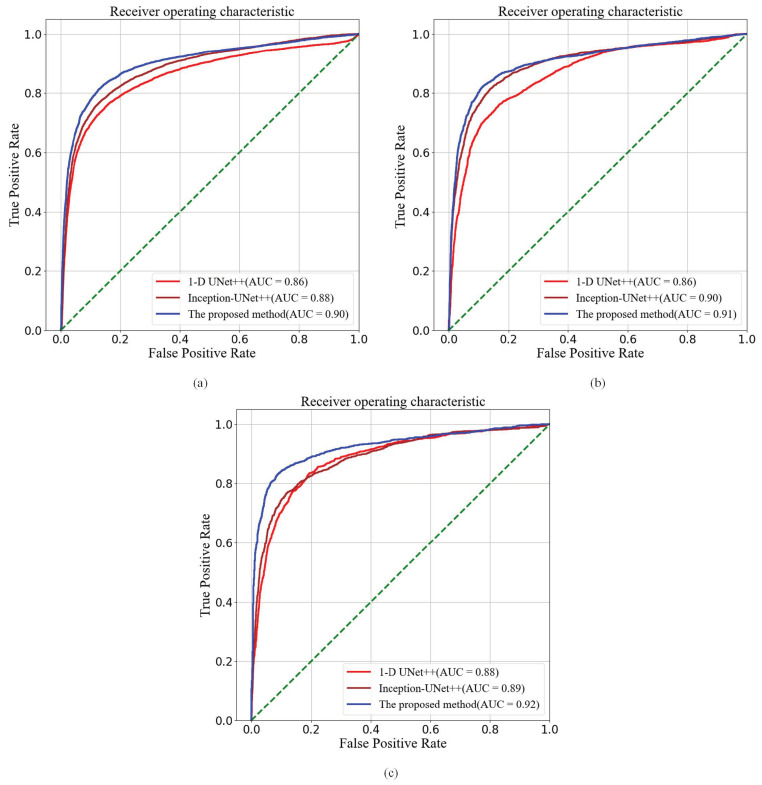
The ROC curve of CHF detection. (**a**) The RR segment length *N* = 500; (**b**)The RR segment length *N* = 1000; (**c**) The RR segment length *N* = 2000.

Except for the four aforementioned performance metrics, Receiver Operating Characteristic (ROC) curve and Area Under ROC Curve (AUC) [32] are also employed to evaluate the CHF detection performance. The ROC curve is able to accurately reflect the relationship between true positive rate (TPR) and the false positive rate (FPR) in a graphical way and is a comprehensive representative of the detection accuracy. AUC is obtained by summing the areas of the parts under the ROC curve. As can be seen from Figure 5, the UNet++ with SE residual modules, which is blue line, achieve the highest AUC values. The corresponding AUC is 0.90, 0.91 and 0.92 on *N* = 500, 1000 and 2000 respectively. Such experimental results demonstrate that the proposed model yields outstanding performance. In addition, the ROC curves of the UNet++ model without SE modules and inception UNet++ are shown in red and brown, respectively.

As shown in Figure 5, the AUC values of 1-D UNet++ without SE modules (AUC = 0.86, 0.86, and 0.88 when *N* = 500, 1000 and 2000, respectively) are less than those of the UNet++ with SE residual modules (AUC = 0.90, 0.91, and 0.92 when *N* = 500, 1000 and 2000, respectively). It mainly owns to that the UNet++ model with SE residual modules can efficiently exploit the information from RR interval segments. SE modules is able to learn the channel-wise calibration and help alleviate the dependencies among channel-wise features. Besides, the skip connection in SE residual blocks is conductive to the back-propagation of gradients and mitigates the declining-accuracy phenomenon in the deep network [35]. The inception modules can improve the expression ability of network by organizing information across channels. Both Table 2 and Figure 5 show that the UNet++ with inception modules do improve the performance in comparing with the plain UNet++ model whereas it is less effective than the improved UNet++ model with SE residual modules.

### 3.3. Comparison with State-of-the-Art CHF Diagnosis Methods

Over the past years, there are a variety of automatic classifiers to diagnose patients with CHF (Table 3). In this experiment, the proposed method is compared with several state-of the-art CHF diagnosis methods, including the methods based on Inception-V4 [20], LSSVM [36], SVM [15], Ensmeble classifier [16] and LSTM [21] in terms of the diagnosis accuracy. Li et al. [20] obtained 81.85% with 300 length RR interval segments. Sharma et al. [36] achieved the accuracy of 87.15% by using *N* = 2000 length RR intervals for the classification of normal and CHF signals. Liu et al. [15] studied CHF detection and obtained the performance of 85.5% and 85.6% on *N* = 1000 and 2000 length RR intervals, respectively. Wang et al. [16] combined expert features of RR intervals with deep-learning features, and fed into the ensemble classifier to differentiate CHF patients from healthy controls. They yielded 83.84%, 87.54% and 85.71% accuracy on *N* = 500, 1000 and 2000 length RR intervals, respectively. Wang et al. [21] presented an LSTM-based inception module to detect CHF and achieved 82.51%, 86.68% and 87.55% accuracy on *N* = 500, 1000 and 2000 length RR intervals, respectively.

**Figure 6:**
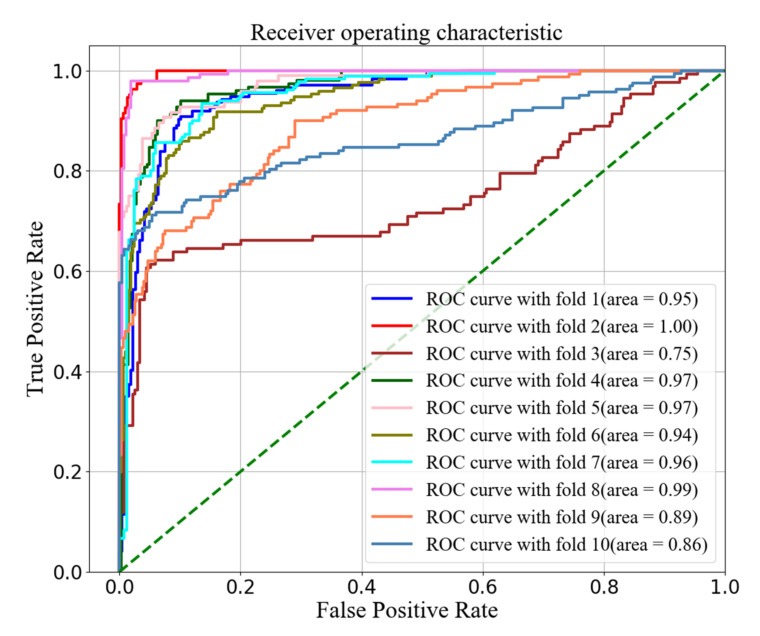
The ROC curve of 10 folds with 2000 sample length.

In [21], Wang et al. employed the traditional 10-fold cross validation and achieved the mean accuracy of 86.42%, 87.76% and 86.63% with *N* = 500, 1000 and 2000 length RR intervals, respectively. However, the shuffled signals of all the patients were divided them into training set and test set. Such division method is against the inter-patient experiment of Association for the Advancement of Medical Instrumentation (AAMI) standard [37]. To overcome such issue, they also employed the blindfold testing to evaluate the result [21]. They randomly selected the RR intervals of 12 subjects as the test data, and achieved an accuracy of 82.51%, 86.68% and 87.55% when *N* = 500, 1000 and 2000 respectively. In this study, to demonstrate how well the model perform on unseen data, we show the ROC curves corresponding to 10 folds when 2000 intervals are employed, as in Figure 6. It is clear that the AUCs vary greatly from fold to fold. The largest AUC is 1 with accuracy of 95.95% (fold 2) whereas the smallest AUC is only 0.75 with accuracy of 78.54% (fold 3).

Compared with other methods, the proposed model achieves the best performance of 85.64%, 86.65% and 88.79% when *N* = 500, 1000 and 2000, respectively. One potential reason is that the proposed method is able to extract more reliable signal features in high dimensional space. UNet ++ shortens the information gap between encoder and decoder through the information fusion between different layers, which makes full use of RR signals. Furthermore, the SE residual blocks is able to emphasize the salient features and suppress the irrelevant information [22].

### 3.4. Performance Evaluation in More Practical Scenario

In real-world applications, clinicians need to differentiate CHF subjects with non-CHF subjects, rather than only the normal subjects. Therefore, to fairly demonstrate the performance of the proposed method in realistic scenario, other types of heart rhythm abnormalities should be considered. In this study, we further evaluate the performance when the RR intervals of atrial fibrillation (AF) patients are also employed as the non-CHF signals. These ECG signals are from public-available long-term AF database which includes 84 long-term (24-h) ECG recordings [38]. After pre-processing, 4333 RR segments of AF (when the segment length *N* = 2000) are obtained. To avoid the problem of class imbalance, we mixed the signals from the Normal Sinus Rhythm (NSR) RR interval Database and long-term AF database, and then randomly selected 2800 RR segments from them. Table 4 illustrates the details of these datasets.

As shown in Table 5, the average accuracy after 70 epochs is 89.33% when the RR intervals of both NSR and AF subjects are utilized as the non-CHF data. The result is similar to that in differentiating NSR and CHF patients. However, this study is a preliminary attempt to automatically diagnose CHF, and many other types of heart rhythm abnormalities will be considered in the future research.

## 4. Discussion and Conclusions

In this work, an automatic classifier for CHF diagnosis via short length HRV signals is proposed. In comparison with previous CHF detection methods, the developed method employs an end-to-end deep learning model to extract features and make decision. To be more specific, the improved 1-D UNet++ architecture involves a residual block to distinguish CHF patients from normal subjects as well as a SE block to highlight the useful features and suppress the useless information. Such classification model obtains information from HRV signals with minimal information reduction and provides the optimal feature of the input RR interval segments. The proposed model outperformed the previous CHF diagnosis with a state-of-art accuracy of 85.64%, 86.65% and 88.79% when 500, 1000 and 2000 RR intervals are employed, respectively. This pilot study demonstrates that the deep learning-based automatic diagnosis can be an important tool to assist clinicians in making wise decisions. Moreover, CHF diagnosis via short-term RR intervals can be transplanted to mobile devices like smartphones easily. It contributes to monitoring the changes of cardiac autonomic nervous function with CHF patients.

Although the proposed method has provided promising results, there are still a few limitations to overcome. First, more training data, especially from many other types of heart rhythm abnormalities, is required to provide more reliable diagnosis. Second, CHF is categorized into four stages by the American College of Cardiology Foundation [39]. The proposed method can only determine whether the patients suffer from CHF or not, but cannot determine the precise CHF stage due to limited numbers of subjects available in stage I and II CHF. 

## Figures and Tables

**Table 1:** The number of signals for different database and classes.

Database	Pre-Processing	Total Segments		
		500	1000	2000
**CHF-RR**	None	6635	3317	1658
	Removing the RR intervals longer than 2 s	6622	3311	1655
	Removing the RR intervals marked as abnormal heartbeats	6271	3129	1558
**NSR-RR**	None	11,555	5777	2888
	Removing the RR intervals longer than 2 s	11,538	5769	2884
	Removing the RR intervals marked as abnormal heartbeats	11,314	5641	2808

**Table 2:** The overall performance with different length of RR segments.

Methods	Segment Length	Evaluation			
		Recall	Precision	F1-Score	Accuracy
**The 1-D UNet++**	500	0.6812	0.7927	0.7248	0.8257
	1000	0.6621	0.7980	0.7176	0.8184
	2000	0.6617	0.8202	0.7279	0.8269
**The inception 1-D UNet++**	500	0.6978	0.8255	0.7488	0.8412
	1000	0.7247	0.8359	0.7684	0.8521
	2000	0.7190	0.8122	0.7529	0.8442
**The proposed method**	500	0.7381	0.8346	0.7793	0.8564
	1000	0.7596	0.8488	0.7947	0.8665
	2000	0.8018	0.8685	0.8281	0.8879

**Table 3:** Comparison of the proposed method against existing methods on CHF detection.

Author (Year)	Classifier	Features	Length	Accuracy
Li (2018) [20]	Inception-V4	Fuzzy GMEn	300	81.85%
Sharma (2018) [36]	LS-SVM	k-NN entropy and correntropy	2000	87.15%
Liu (2017) [15]	SVM	Multiscale entropy of RR	1000	85.5%
			2000	85.6%
Wang (2019) [16]	Ensemble classifier	Expert features and deep-learning features	500	83.84%
			1000	87.54%
			2000	85.71%
Wang (2019) [21]	LSTM based Inception	-	500	82.51%
			1000	86.68%
			2000	87.55%
Our proposed method	Improved UNet++	-	500	85.64%
			1000	86.65%
			2000	88.79%

**Table 4:** The number of ECG recordings in each dataset when the segment length *N* = 2000.

Classes	Database	Total Segments (*N* = 2000)
Non-CHF	NSR RR interval Database	2808
	Long-Term AF Database	4333
CHF	After random sampling	2800
	CHF RR interval Database	1558

**Table 5:** The overall performance with different kinds of non-CHF signals when the segment length *N* = 2000.

Data	Evaluation			
	Recall	Precision	F1-Score	Accuracy
CHF vs. NSR	80.18%	86.85%	82.81%	88.79%
CHF vs. NSR and AF	78.67%	88.26%	82.24%	89.33%

## Data Availability

Not applicable.
